# From bacterial to human dihydrouridine synthase: automated structure determination

**DOI:** 10.1107/S1399004715009220

**Published:** 2015-06-30

**Authors:** Fiona Whelan, Huw T. Jenkins, Samuel C. Griffiths, Robert T. Byrne, Eleanor J. Dodson, Alfred A. Antson

**Affiliations:** a Department of Biology, The University of York, Heslington, York YO10 5DD, England; b York Structural Biology Laboratory, Department of Chemistry, The University of York, Heslington, York YO10 5DD, England; c Division of Structural Biology, University of Oxford, Headington, Oxford OX3 7BN, England; d Gene Center and Department of Biochemistry, Ludwig-Maximilians-University Munich, Feodor-Lynen-Strasse 25, 81377 Munich, Germany

**Keywords:** dihydrouridine synthase, X-ray crystallography, *MR-Rosetta*, tRNA modification, lung cancer

## Abstract

The reduction of uridine to dihydrouridine at specific positions in tRNA is catalysed by dihydrouridine synthase (Dus) enzymes. Increased expression of human dihydrouridine synthase 2 (hDus2) has been linked to pulmonary carcinogenesis, while its knockdown decreased cancer cell line viability, suggesting that it may serve as a valuable target for therapeutic intervention. Here, the X-ray crystal structure of a construct of hDus2 encompassing the catalytic and tRNA-recognition domains (residues 1–340) determined at 1.9 Å resolution is presented. It is shown that the structure can be determined automatically by *phenix.mr_rosetta* starting from a bacterial Dus enzyme with only 18% sequence identity and a significantly divergent structure. The overall fold of the human Dus2 is similar to that of bacterial enzymes, but has a larger recognition domain and a unique three-stranded antiparallel β-sheet insertion into the catalytic domain that packs next to the recognition domain, contributing to domain–domain interactions. The structure may inform the development of novel therapeutic approaches in the fight against lung cancer.

## Abstract 2

The crystal structure of a human dihydrouridine synthase, an enzyme associated with lung cancer, with 18% sequence identity to a *T. maritima* enzyme, has been determined at 1.9 Å resolution by molecular replacement after extensive molecular remodelling of the template.

## 1. Introduction   

**Figure 1:**
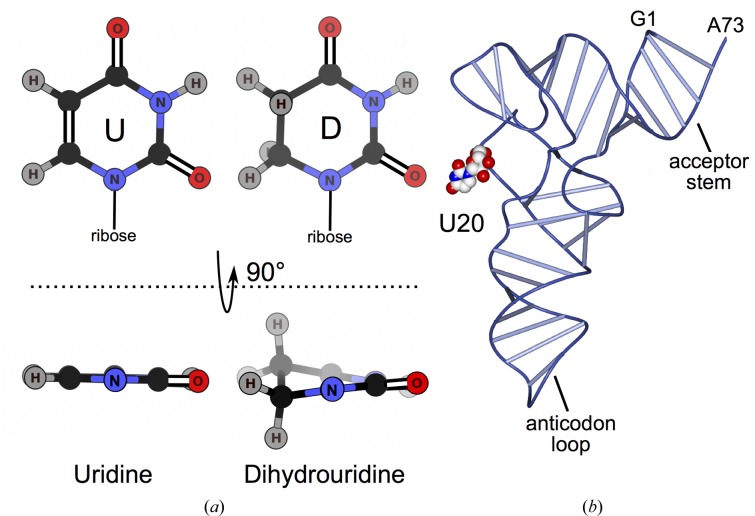
Dihydrouridine modification. (*a*) Uridine and dihydrouridine. (*b*) U20 location shown for *E. coli* tRNA^Phe^ (Byrne *et al.*, 2010 ▸). U20 is illustrated by a van der Waals model coloured by atom type with O atoms in red, N atoms in blue and C atoms in white.

**Figure 2:**
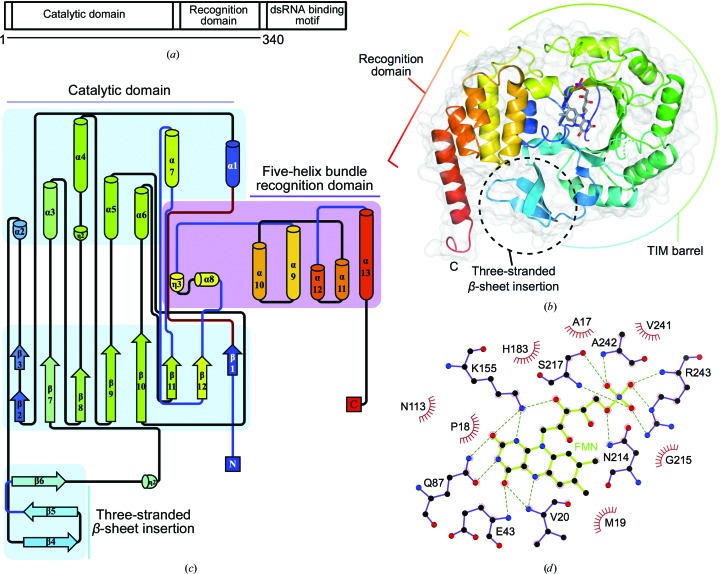
The structure of hDus2 1–340 comprising the catalytic and recognition domains. (*a*) Domain schematic of hDus2. (*b*) The structure of hDus2 viewed from the putative tRNA-binding surface. The ribbon diagram is coloured from blue (N-terminus) to red (C-terminus); missing residues 118–128 are indicated by a dashed green ribbon; bound FMN is shown as sticks. (*c*) Secondary-structure topology diagram [colouring as in (*b*)] showing domain composition and connectivity. (*d*) FMN coordination, with FMN and active-site residues shown as sticks, hydrogen bonds shown as dashed lines, atoms coloured according to type and residues forming hydrophobic contacts designated by red semicircles.

tRNA is extensively modified, with 105 different nucleoside modifications identified to date (Cantara *et al.*, 2011 ▸). Modifications are catalyzed enzymatically during post-transcriptional maturation of tRNA. They occur at specific positions, in about 10% of nucleosides in total (Jühling *et al.*, 2009 ▸), altering local chemistry and affecting tRNA conformation and flexibility (Dalluge *et al.*, 1996 ▸; Motorin & Helm, 2010 ▸). tRNA modifications have been shown to affect human health (Nallagatla *et al.*, 2013 ▸; Gehrig *et al.*, 2012 ▸) and are associated with disorders including cancer (Chen *et al.*, 2009 ▸; Rakovich *et al.*, 2011 ▸; Zinshteyn & Gilbert, 2013 ▸; Kato *et al.*, 2005 ▸; Kuchino & Borek, 1978 ▸; Begley *et al.*, 2013 ▸; Spinola *et al.*, 2005 ▸). One of the most common modified nucleosides, dihydrouridine (D) is produced by dihydrouridine synthase (Dus) by enzymatic reduction of the C_5_—C_6_ bond in uridine (U) (Fig. 1 ▸ *a*). The nonplanar base of dihydrouridine is unable to form stacking interactions with bases of other nucleosides, increasing flexibility (Dalluge *et al.*, 1996 ▸). Dihydrouridine has been postulated to aid in the fidelity of translation and to define cognate interactions with partner aminoacyl-tRNA synthetases (Hendrickson, 2001 ▸ and references therein). Given that dihydrouridine increases flexibility, its incorporation has been proposed to be more important for tRNA folding in psychrophiles and mesophiles (Dalluge *et al.*, 1997 ▸). *Escherichia coli* has three Dus enzymes that perform specific, nonredundant modifications (Bishop *et al.*, 2002 ▸). In humans, there are four Dus enzymes. The hDus2 subfamily is proposed to specifically modify U20 based on sequence identity and biochemical data available for *Saccharomyces cerevisiae* Dus2 (Xing *et al.*, 2004 ▸). hDus2 contains 493 residues and comprises an N-terminal catalytic domain, a central tRNA-recognition domain and a C-terminal dsRNA-binding domain (369–433) (Fig. 2 ▸ *a*). There is growing evidence that overexpression of hDus2 potentiates the growth of non-small cell lung carcinoma (NSCLC; Kato *et al.*, 2005 ▸). Elevated hDus2 mRNA and protein levels were identified in a range of NSCLC cell lines, and siRNA-dependent knockdown of hDus2 decreased colony formation and cell viability, while hDus2 immuno­histochemical staining correlated with patient survival and was also defined as an independent prognostic factor for the development of NSCLC (Kato *et al.*, 2005 ▸). Additionally, increased levels of dihydrouridine incorporation have been identified in other mammalian carcinomas (Kuchino & Borek, 1978 ▸).

X-ray structures are available for three Dus enzymes. The first structure to be solved was of the TM0096 protein from *Thermotoga maritima* (PDB entry 1vhn; Park *et al.*, 2004 ▸), which has not yet been functionally characterized. The second was of the position 20 specific enzyme from *Thermus thermophilus* (*Tt*Dus; PDB entry 3bop; Yu *et al.*, 2011 ▸). For the third enzyme, the position-16 specific *E. coli* DusC, two structures are available at a higher resolution (*Ec*DusC, PDB entry 4bfa; Byrne *et al.*, 2015 ▸) and a lower resolution (PDB entry 3w9z; Chen *et al.*, 2013 ▸). All three enzymes have a conserved catalytic domain comprising a TIM barrel with a bound flavin mononucleotide (FMN) and a C-terminal α-helical tRNA-recognition domain that varies slightly in its relative orientation to the TIM barrel. Bound FMN functions as a cofactor during the reduction of the C_5_—C_6_ double bond of uridine (Rider *et al.*, 2009 ▸). For *Tt*Dus and *Ec*DusC, structural information is also available for complexes with tRNA indicating that the orientation of the tRNA substrate defines the modification specificity of the enzyme (PDB entries 3b0v, 3b0u, Yu *et al.*., 2011 ▸ and 4yco, 4ycp, Byrne *et al.*, 2015 ▸). The structure of *Tt*Dus covalently bound to target tRNA was determined at 3.5 Å resolution and a higher 1.95 Å resolution structure was obtained for the enzyme cross-linked to a smaller tRNA fragment (PDB entries 3b0v and 3b0u, respectively; Yu *et al.*, 2011 ▸). To date, no structural information on archaeal or eukaryotic Dus has been reported. Here, we present the X-ray structure of a human Dus2 protein construct comprising the catalytic and tRNA-recognition domains.

The rapid growth of the PDB has led to an increasingly large pool of search models for use in molecular replacement. In many cases search models with low identity produce solutions that are correctly placed, but the noisy electron-density maps calculated using phases from the model contain insufficient information to guide improvement of the model. In such cases, recent developments using molecular-modelling tools to rebuild the placed model have produced spectacular results (DiMaio *et al.*, 2011 ▸; DiMaio, 2013 ▸; Terwilliger *et al.*, 2012 ▸). As there are three possible template structures available (*Tm*Dus, *Tt*Dus and *Ec*DusC), we investigated whether any of these could be used to solve the hDus2 structure by automated molecular replacement and rebuilding using *phenix.mr_rosetta*. In order to compare the phasing methods, the phases were also determined experimentally using SAD techniques.

## 2. Methods   

### 2.1. Purification, crystallization and data collection   

Cloning, purification, crystallization and data collection of native hDus2 1–340 was performed as described previously (Griffiths *et al.*, 2012 ▸). Selenomethionyl hDus2 1–340 was expressed and purified in the same manner as the native protein, using immobilized metal-affinity and anion-exchange chromatography, except that *E. coli* B834 (DE3) cells were used for expression and were grown in minimal medium supplemented with 40 µg ml^−1^ l-selenomethionine (Hendrickson *et al.*, 1990 ▸). Labelling was confirmed using matrix-assisted laser desorption/ionization mass spectrometry (MALDI-MS) and the protein (at 10 mg ml^−1^ in 20 m*M* Tris, 100 m*M* NaCl, 5 m*M* imidazole, 5 m*M* DTT pH 8) was subjected to crystallization screening using the PACT screen (Newman *et al.*, 2005 ▸) in 0.3 µl sitting drops with 54 µl reservoir incubated at 292 K. Rhomboid-shaped crystals grew in 2 days from a condition comprising 0.1 *M* MES–malic acid–Tris pH 4, 25%(*w*/*v*) PEG 1500 (Newman, 2004 ▸). Cryoprotection was performed by passing crystals through reservoir solution supplemented with 200 m*M* NaCl, 40 m*M* Tris pH 8 and 32% PEG 1500 prior to flash-cooling in liquid N_2_. X-ray data for selenomethionyl hDus2 were collected at 100 K on the I04 beamline at Diamond Light Source (DLS), Didcot, England using an ADSC Q315r CCD detector. Indexing and integration was performed with *XDS* (Kabsch, 2010 ▸). *POINTLESS* (Evans, 2011 ▸) was used to confirm the Laue group and *AIMLESS* was used for scaling and merging (Evans & Murshudov, 2013 ▸). X-ray data statistics are summarized in Table 1 ▸.

### 2.2. Initial structure determination   

The structure was first determined by single-wavelength anomalous diffraction (SAD) using selenomethionyl protein. Selenium sites were identified using *SHELXC*/*SHELXD* (Sheldrick, 2008 ▸), phases were calculated and refined using *Phaser* (McCoy *et al.*, 2007 ▸) and density modification was performed with *Parrot* (Cowtan, 2010 ▸). *Buccaneer* (Cowtan, 2006 ▸) was deployed for initial automated model building. Subsequent manual rebuilding was performed with *Coot* (Emsley & Cowtan, 2004 ▸; Emsley *et al.*, 2010 ▸) and the model was refined using *REFMAC*5 (Murshudov *et al.*, 2011 ▸). The structure of the native protein was then determined at a higher resolution by molecular replacement using *MOLREP* (Vagin & Teplyakov, 2010 ▸). This model was refined using isotropic *B* factors and four TLS groups (residues 7–109, 110–220, 221–319 and 320–339) defined by the *TLS Motion Determination* server (Painter & Merritt, 2006 ▸; Table 1 ▸). The coordinates and structure factors have been deposited in the Protein Data Bank and are available under accession code 4xp7.

### 2.3. Exploring the power of molecular replacement using distant homologues   

For automated molecular replacement and rebuilding from low-identity templates, we used *phenix.mr_rosetta* (DiMaio *et al.*, 2011 ▸; DiMaio, 2013 ▸; Terwilliger *et al.*, 2012 ▸). A sequence alignment of hDus2 residues 1–340 with *Tt*Dus (PDB entry 3b0p), *Tm*Dus (PDB entry 1vhn) and *Ec*DusC (PDB entry 4bfa) was generated using *HHpred* (Söding *et al.*, 2005 ▸). Fragments were generated from the sequence of hDus2 using the *Robetta* fragment server (Kim *et al.*, 2004 ▸). The alignment, fragment files from the *Robetta* fragment server (http://robetta.bakerlab.org/), the native data set and the sequence of hDus2 residues 1–340 were used as input for *phenix.mr_rosetta* executed on a single computer. 100 *Rosetta* models were built for each MR solution, taking between 50 and 75 CPU hours. As the best solution from the final *phenix.autobuild* step of the first round is (by default) rebuilt again with *Rosetta*, a run of *phenix.mr_rosetta* took around 100–150 CPU hours to generate a final model from each molecular-replacement solution. The density-modified phases and FOMs from the highest scoring cycle of *phenix.autobuild* in the final stage of *phenix.mr_rosetta* for each of the three templates were used for model building with *Buccaneer* (Cowtan, 2006 ▸, 2008 ▸) and refinement with *REFMAC*5 (Murshudov *et al.*, 2011 ▸).

In order to compare the quality of the three models produced by these automated (*i.e.* no manual building) methods with that produced by experimental phasing, the *Buccaneer* model built into the experimentally phased map (as described previously) was used as a MR search model with the (higher resolution) native data. The placed model was used to generate density-modified phases with *RESOLVE via phenix.autobuild* (Terwilliger, 2000 ▸; Terwilliger *et al.*, 2008 ▸). These density-modified phases and FOMs were combined with the native data and used for model building as described above.

For a conventional MR approach, the sequences of hDus2 1–340 and the search models *Tt*Dus (PDB entry 3b0p), *Tm*Dus (PDB entry 1vhn) and *Ec*DusC (PDB entry 4bfa) were aligned by *HHpred* (Söding *et al.*, 2005 ▸). Sequences were trimmed to the catalytic domain; search models were truncated and edited using *Sculptor* (Bunkóczi & Read, 2011 ▸). *Phaser* (McCoy *et al.*, 2007 ▸) solutions with the highest LLG were refined by *REFMAC*5 using the jelly-body option and autobuilt using *Buccaneer*.

### 2.4. Sequence conservation and structure superposition   

Structure-based alignment of the hDus2, *S. cerevisiae* Dus2, *S. pombe* Dus2, *Tt*Dus, *Ec*DusC and *Tm*Dus sequences (UniProt IDs Q9NX74, P53720, O74731, Q5SMC7, P33371 and Q9WXV1, respectively) was performed using the *Expresso* server (Armougom *et al.*, 2006 ▸) and presented using *ESPript* (Robert & Gouet, 2014 ▸). Sequences within the eukaryotic Dus2 subfamily were aligned using *Clustal Omega* and conservation was mapped onto the protein surface by *ConSurf* (Landau *et al.*, 2005 ▸) and rendered using *CCP*4*mg* (McNicholas *et al.*, 2011 ▸). Sequence alignment of representative members of the three eukaryotic subfamilies including Dus2, Dus1 and Dus4 was performed using *Clustal Omega* and presented using *ESPript*. The Dus3 subfamily was not included in this alignment owing to significant sequence differences (Kasprzak *et al.*, 2012 ▸). The structures of *Tt*Dus, *Tm*Dus, *Ec*DusC (PDB entry 4bfa) and hDus2 were aligned by secondary-structure matching using *Superpose* (Krissinel & Henrick, 2004 ▸) and the cartoons were rendered with *CCP*4*mg* (McNicholas *et al.*, 2011 ▸). Electrostatic surfaces were calculated with *PDB*2*PQR* (Dolinsky *et al.*, 2004 ▸) and *APBS* (Baker *et al.*, 2001 ▸) and were rendered by *PyMOL* (v.1.3r1; Schrödinger). Cofactor-coordination figures were generated using *LigPlot+* (Laskowski & Swindells, 2011 ▸) and the protein-topology two-dimensional cartoon was generated by *Pro-Origami* (Stivala *et al.*, 2011 ▸).

## 3. Results and discussion   

### 3.1. Initial structure determination by SAD   

The structure of hDus2 was initially determined by the conventional SAD approach using crystals of selenomethionyl protein that diffracted to 2.3 Å resolution. Following this, we also attempted to solve this structure by MR with distant sequence homologues using *phenix.mr_rosetta* (DiMaio *et al.*, 2011 ▸; Terwilliger *et al.*, 2012 ▸) and the 1.9 Å resolution native data set. The results of the structure-determination attempts are described below.

The crystals of selenomethione-labelled hDus2, unlike the *P*2_1_ crystals of the native protein, belonged to space group *C*2 (Table 1 ▸). There is one molecule per asymmetric unit, corresponding to a solvent content of 27% (Matthews, 1968 ▸). Density-modified SAD phases resulted in clear electron-density maps and autobuilding was able to produce an almost complete model. The FMN density was nonplanar, hence the two-electron-reduced form (PDB ligand ID FNR; Supplementary Fig. S1) was used during the refinement (Table 1 ▸).

### 3.2. *Post hoc* structure determination by molecular replacement   

Previous attempts at rebuilding potential MR solutions obtained with the *Tm*Dus and *Ec*DusC models were unsuccessful. Indeed, autobuilding with *phenix.autobuild* or density modification with *RESOLVE* followed by extensive rounds of autobuilding with either *Buccaneer* or *ARP*/*wARP* (Langer *et al.*, 2008 ▸) starting from the models placed in the initial MR stage of *phenix.mr_rosetta* (Supplementary Fig. S2*a*) failed to produce solutions with an *R*_free_ of less than 50%. As the sequence alignment generated with *HHpred* contains residues 1 to 340 of hDus2, the search models used in *phenix.mr_rosetta* contain parts of the recognition domains of *Tt*Dus, *Tm*Dus and *Ec*DusC that align with the recognition domain of hDus2. The *Phaser* translation-function *Z*-score (TFZ) and LLG for the top solutions were relatively poor: *Tt*Dus, TFZ 4.7, LLG 39.7; *Tm*Dus, TFZ 5.2, LLG 26.0; *Ec*DusC, TFZ 4.8, LLG 25.6. Correlation between the 2*mF*_o_ − *DF*_c_ maps calculated from these solutions and the map calculated from the final refined structure of hDus2 was ∼0.35 for *Tt*Dus and *Tm*Dus and ∼0.30 for *Ec*DusC (Supplementary Table S1). It is therefore unsurprising that autobuilding from these MR solutions was not possible. However, despite the low sequence identity to *Tt*Dus, *Tm*Dus and *Ec*DusC (20.3, 17.9 and 22.6%, respectively), *phenix.mr_rosetta* was able to generate excellent maps for hDus2 using *Tt*Dus and *Tm*Dus as search models. Autobuilding into these maps with *Buccaneer* produced almost complete models (Supplementary Fig. S2*b*, Supplementary Table S2).

Despite having the highest sequence identity to hDus2, *Ec*DusC proved to be the poorest search model, as demonstrated by the lowest correlation between maps calculated from this model and the final refined structure, and *phenix.mr_rosetta* failed to generate a map suitable for autobuilding. However, during the revision stage of this manuscript we investigated whether the latest versions of *PHENIX* and *Rosetta* would be able to rebuild from the MR solution obtained for the *Ec*DusC model, which was clearly correctly placed in the unit cell. It transpired that the current versions are able to improve this model and generate maps suitable for autobuilding and refinement using the same versions of *Buccaneer*/*REFMAC*5 as previously (Supplementary Fig. S2*b*, Supplementary Table S2).

Given the success of *phenix.mr_rosetta*, we investigated whether it was possible to solve the structure of hDus2 by more conventional approaches without remodelling by *Rosetta*. Superposition of *Tt*Dus, *Tm*Dus and *Ec*DusC revealed a highly similar catalytic domain but variation in the conformation and the relative position of the C-terminal recognition domain (Supplementary Fig. S2*c*). Sequence alignments with hDus2 indicated homology in the catalytic domain (Supplementary Fig. S3). Hence, search models were generated using *Sculptor* based on the *HHpred* alignments used as input for *phenix.mr_rosetta* but encompassing only the catalytic domain. These minimal catalytic domain structures were used as search models with *Phaser*. A clear solution (TFZ 8.4, LLG 83.4) was found for the *Tt*Dus catalytic domain model, with a 2*mF*_o_ − *DF*_c_ map correlation of 0.37 to the map calculated from the final refined structure of hDus2 (Supplementary Table S3). 100 cycles of *REFMAC* jelly-body refinement resulted in an *R* and *R*_free_ of 46.4 and 48.3%, respectively, and a map correlation of 0.56. Iterative rebuilding and refinement of this model with *Buccaneer* and *REFMAC*5 generated a near-complete model of hDus2 with an *R*_free_ of <30%. Attempts to repeat this approach with the *Tm*Dus and *Ec*DusC catalytic domains as search models produced reasonable map correlation after jelly-body refinement only for *Tm*Dus (Supplementary Table S3). Whilst rebuilding the *Tm*Dus solution to a complete model of hDus2 was possible, it required extensive iteration of jelly-body refinement and *Buccaneer* rebuilding/*REFMAC* refinement. In agreement, superposition of the catalytic domains shows that hDus2 is most similar to *Tt*Dus (C^α^ r.m.s.d. of 1.5 Å for residues 3 to 240). In contrast, *Tm*Dus (C^α^ r.m.s.d. of 2.0 Å for residues 5–228) and *Ec*DusC (C^α^ r.m.s.d. of 1.9 Å for residues 1–240) were more divergent (Supplementary Fig. S2*d*, Supplementary Table S3).

### 3.3. Unique features in the fold and relative orientation of hDus2 domains   

**Figure 3:**
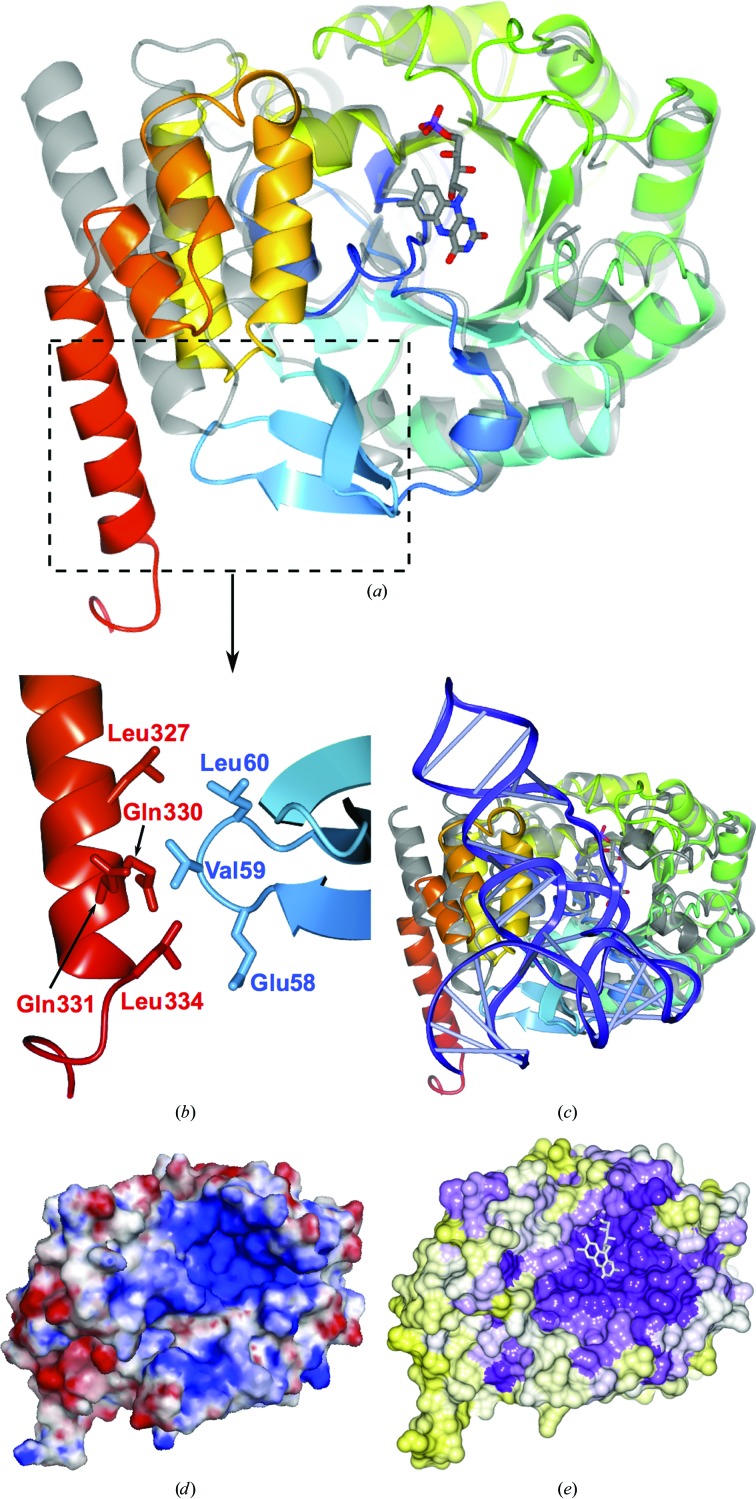
Comparison of hDus2 with *Tt*Dus and analysis of electrostatic surface and sequence conservation. (*a*) Ribbon diagram showing superposition of hDus2 with *Tt*Dus (grey). The boxed area is magnified in (*b*), showing hDus2 interactions between the catalytic and recognition domains that are not present in bacterial Dus enzymes. (*c*) hDus2 superposed with the structure of the *Tt*Dus–tRNA complex (with *Tt*Dus in grey and tRNA in blue). (*d*) hDus2 molecular surface coloured according to electrostatic potential (±5*kT*/e; red, negative; blue, positive). (*e*) hDus2 surface coloured according to sequence conservation: high (purple) to low (yellow).

Like other Dus enzymes, hDus2 folds into catalytic and tRNA-recognition domains (Figs. 2 ▸ *b* and 2 ▸ *c*). The catalytic domain, containing the bound FMN cofactor (Fig. 2 ▸ *d*), comprises residues 7–258 of the protein, being roughly two times larger than the recognition domain (residues 259–339). Superposition of hDus2 with *Tt*Dus illustrates that the enzymes are broadly similar (C^α^ r.m.s.d. of 2.8 Å, 239 residues aligned; Fig. 3 ▸ *a*). There are two major differences between hDus2 and its bacterial enzyme homologues. Firstly, the catalytic domain contains a three-stranded antiparallel β-sheet insertion (residues 53–76; β4–β5–β6; Figs. 2 ▸ *b* and 2 ▸ *c* and Supplementary Fig. S4*a*). Secondly, the recognition domain is larger, comprising a five-helix bundle with a considerably longer C-terminal helix which is positioned differently with respect to the catalytic domain (Fig. 3 ▸ *a* and Supplementary Fig. S4*b*).

Superposition of hDus2 and *Tt*Dus (C^α^ r.m.s.d. of 1.5 Å for the catalytic domain) reveals differences at the interface between the catalytic and recognition domains (Fig. 3 ▸ *b*). The different orientation of the recognition domain in hDus2 results in helices α10 and α11 extending into the area occupied by tRNA in *Tt*Dus (Fig. 3 ▸ *c*). The position of the recognition domain is stabilized by the three-stranded β-sheet insertion in the catalytic domain, which packs next to the recognition domain. The β4–β5 loop (residues 58–60) of the β-sheet interacts with residues 327–334 of the recognition domain (Fig. 3 ▸ *b*). In addition to mediating interactions between the two domains, the β-sheet insertion extends into the tRNA-occupied area of *Tt*Dus (Fig. 3 ▸ *c* and Supplementary Fig. S4*c*), suggesting a role in protein–tRNA interactions. It is interesting to note that the three-stranded β-sheet insertion appears to be unique to the eukaryotic Dus2 subfamily (Supplementary Fig. S5).

### 3.4. tRNA binding and the active site   

*Tt*Dus makes contacts with the tRNA T-loop through helix α6 (residues 96–101). The corresponding segment of hDus2 (residues 118–128) is not defined in the electron-density maps. *Tt*Dus also forms hydrogen bonds to the target residue U20 and to G44 in the variable-loop region *via* Lys175 and Arg178 of the 3_10_-helix (residues 174–180). This helix is not present in hDus2, nor is there any significant sequence conservation in this segment (Supplementary Fig. S3), indicating there may be differences in the manner that hDus2 binds its target tRNA substrates.

hDus2 has a positively charged surface area (Fig. 3 ▸ *d*) which appears to be most similar to the positively charged area of *Tt*Dus, but its size is smaller than in bacterial enzymes (Supplementary Fig. S6). Proximal to the catalytic Cys116 is a loop which was not visible in the electron density (residues 118–128). Some decrease in the positively charged area may be due to two lysine residues present in this loop. Significantly, the positively charged area shows high sequence conservation (Fig. 3 ▸ *e*), indicating functional importance. Part of the positively charged and conserved area of hDus2 corresponds to the active site containing the bound FMN molecule. The residues coordinated to the FMN are highly conserved in *Tt*Dus, *Tm*Dus and *Ec*DusC (Fig. 2 ▸ *d*, Supplementary Figs. S3 and S7), indicating a similar enzymatic mechanism. In the structures of *Tt*Dus and the lower resolution structure of *Ec*DusC (PDB entry 3w9z; Chen *et al.*, 2013 ▸), positive difference density in the active site was attributed to an unknown cofactor, which was modelled as a sulfate moiety in the *Tm*Dus model. Notably, no equivalent density is observed in the difference electron-density maps of hDus2.

## 4. Conclusions   

We have determined the structure of the human tRNA-modification enzyme hDus2 that has been implicated in lung cancer (Kato *et al.*, 2005 ▸). The structure differs in two major ways from those of bacterial Dus enzymes. Firstly, the catalytic domain contains an additional three-stranded β-sheet that is absent from the bacterial enzymes and forms an interacting surface with the recognition domain. Secondly, the recognition domain, in addition to having an additional α-helix, is positioned differently with respect to the catalytic domain. Interactions between the extended C-terminal helix of the recognition domain and the three-stranded β-sheet of the catalytic domain that were not seen in bacterial Dus enzymes appear to extend the domain interface and may stabilize the overall structure. Conservation of the catalytic residues indicates that the enzymatic mechanism is essentially the same as for the bacterial homologues. We propose that structural differences and differences in the electrostatic surface may result in altered positioning of tRNA during catalysis compared with bacterial enzymes. Whilst a complete understanding of tRNA recognition and modification awaits the determination of the X-ray structure of a complex of hDus2 with tRNA, the structural data presented here will inform the design of potent inhibitors of the enzyme.

In spite of the low sequence identity to available search models, automatic approaches using *phenix.mr_rosetta* were successfully used *post hoc* to determine the structure of hDus2, demonstrating that the structures of functional homologues, despite having sequence identities below 20%, can be used for structure determination by molecular replace­ment. Whilst it was in fact possible to solve the structure of hDus2 without the use of *phenix.mr_rosetta*. this required extensive manual intervention that included refinement and model rebuilding. Counterintuitively, the highest identity search model proved to be the worst template, complicating structure determination. In contrast, the use of *phenix.mr_rosetta*, while computationally expensive, required no intervention from the user to achieve a near-complete model. Retrospective analaysis suggested that it was not necessary to build as many as 100 *Rosetta* models for each solution. Indeed, by taking only the top MR solution for each of the three templates and building 20 *Rosetta* models for each solution a complete model can be produced in ∼25 h on a modest four-core desktop computer. Such an automated approach can liberate time for other ‘bottlenecks’ of biological crystallo­graphy associated with protein production and obtaining diffraction-quality crystals.

## Supplementary Material

PDB reference: human dihydrouridine synthase, 4xp7

Supplementary Figures. DOI: 10.1107/S1399004715009220/rr5097sup4.pptx

Supplementary Tables.. DOI: 10.1107/S1399004715009220/rr5097sup1.pdf

## Figures and Tables

**Table 1:** Data-collection statistics Values in parentheses are for the highest resolution shell.

	Native	Selenomethionyl
Data collection		
X-ray source	Beamline I04, DLS	Beamline I04, DLS
Wavelength ()	0.9795	0.9795
Space group	*P*2_1_	*C*2
Unit-cell parameters (, )	*a* = 46.3, *b* = 49.6, *c* = 69.6, = 95.7	*a* = 73.0, *b* = 50.1, *c* = 72.1, = 90.7
Resolution limits ()	46.01.9 (2.01.9)	36.52.3 (2.42.3)
Unique reflections	24243 (3515)	11728 (1136)
Completeness (%)	97.6 (97.8)	100 (100)
Mutliplicity	3.0 (2.8)	3.8 (3.8)
*I*/(*I*)	10.3 (1.7)	9.5 (2.6)
*R*_merge_ † (%)	6.3 (58.5)	8.4 (40.8)
*R*_p.i.m._ ‡ (%)	4.0 (41.4)	8.0 (38.7)
CC_1/2_ §	0.997 (0.722)	0.992 (0.790)
No. of molecules in asymmetric unit	1	1
*V*_M_ (^3^Da^1^)	2.06	1.68
Solvent content (%)	40.2	26.9
Wilson *B* factor (^2^)	25.5	26.2
Structure refinement		
Resolution range ()	46.01.9	
No. of reflections in refinement	22995	
*R* factor (%)	16.8	
No. of reflections used for *R*_free _	1236	
Free *R* factor (%)	20.7	
No. of protein atoms	2466	
No. of water molecules	194	
*B* factor (^2^)	36.6	
R.m.s.d.		
Bond lengths ()	0.012	
Bond angles ()	1.5	
Ramachandran statistics		
Favoured (%)	98	
Allowed (%)	2	
Disallowed (%)	0	

† *R*_merge_ = 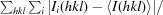 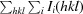.

‡ *R*_p.i.m._ = 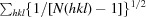 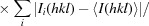 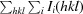.

§ CC_1/2_ is the half-data-set correlation coefficient.

## References

[bb1] Armougom, F., Moretti, S., Poirot, O., Audic, S., Dumas, P., Schaeli, B., Keduas, V. & Notredame, C. (2006). *Nucleic Acids Res.* **34**, W604–W608. 10.1093/nar/gkl092 PMC1538866 16845081

[bb2] Baker, N. A., Sept, D., Joseph, S., Holst, M. J. & McCammon, J. A. (2001). *Proc. Natl Acad. Sci. USA*, **98**, 10037–10041. 10.1073/pnas.181342398 PMC56910 11517324

[bb3] Begley, U., Sosa, M. S., Avivar-Valderas, A., Patil, A., Endres, L., Estrada, Y., Chan, C. T. Y., Su, D., Dedon, P. C., Aguirre-Ghiso, J. A. & Begley, T. (2013). *EMBO Mol. Med.* **5**, 366–383. 10.1002/emmm.201201161 PMC3598078 23381944

[bb4] Bishop, A. C., Xu, J., Johnson, R. C., Schimmel, P. & de Crécy-Lagard, V. (2002). *J. Biol. Chem.* **277**, 25090–25095. 10.1074/jbc.M203208200 11983710

[bb5] Bunkóczi, G. & Read, R. J. (2011). *Acta Cryst.* D**67**, 303–312. 10.1107/S0907444910051218 PMC3069745 21460448

[bb6] Byrne, R. T., Konevega, A. L., Rodnina, M. V. & Antson, A. A. (2010). *Nucleic Acids Res.* **38**, 4154–4162. 10.1093/nar/gkq133 PMC2896525 20203084

[bb62] Byrne, R. T., Jenkins, H. T., Peters, D. T., Whelan, F., Stowell, J., Aziz, N., Kasatsky, P., Rodnina, M. V., Koonin, E. V., Konevega, A. L. & Antson, A. A. (2015). *Proc. Natl Acad. Sci. USA*, **112**, 6033–6037. 10.1073/pnas.1500161112 PMC4434734 25902496

[bb7] Cantara, W. A., Crain, P. F., Rozenski, J., McCloskey, J. A., Harris, K. A., Zhang, X., Vendeix, F. A. P., Fabris, D. & Agris, P. F. (2011). *Nucleic Acids Res.* **39**, D195–D201. 10.1093/nar/gkq1028 PMC3013656 21071406

[bb8] Chen, C., Tuck, S. & Byström, A. S. (2009). *PLoS Genet.* **5**, e1000561. 10.1371/journal.pgen.1000561 PMC2702823 19593383

[bb9] Chen, M., Yu, J., Tanaka, Y., Tanaka, M., Tanaka, I. & Yao, M. (2013). *Acta Cryst.* F**69**, 834–838. 10.1107/S1744309113019489 PMC3729154 23908023

[bb10] Cowtan, K. (2006). *Acta Cryst.* D**62**, 1002–1011. 10.1107/S0907444906022116 16929101

[bb11] Cowtan, K. (2008). *Acta Cryst.* D**64**, 83–89. 10.1107/S0907444907033938 PMC2394793 18094471

[bb12] Cowtan, K. (2010). *Acta Cryst.* D**66**, 470–478. 10.1107/S090744490903947X PMC2852311 20383000

[bb13] Dalluge, J. J., Hamamoto, T., Horikoshi, K., Morita, R. Y., Stetter, K. O. & McCloskey, J. A. (1997). *J. Bacteriol.* **179**, 1918–1923. 10.1128/jb.179.6.1918-1923.1997 PMC178914 9068636

[bb14] Dalluge, J. J., Hashizume, T., Sopchik, A. E., McCloskey, J. A. & Davis, D. R. (1996). *Nucleic Acids Res.* **24**, 1073–1079. 10.1093/nar/24.6.1073 PMC145759 8604341

[bb15] DiMaio, F. (2013). *Acta Cryst.* D**69**, 2202–2208. 10.1107/S0907444913023305 PMC3817693 24189231

[bb16] DiMaio, F., Terwilliger, T. C., Read, R. J., Wlodawer, A., Oberdorfer, G., Wagner, U., Valkov, E., Alon, A., Fass, D., Axelrod, H. L., Das, D., Vorobiev, S. M., Iwaï, H., Pokkuluri, P. R. & Baker, D. (2011). *Nature (London)*, **473**, 540–543. 10.1038/nature09964 PMC3365536 21532589

[bb17] Dolinsky, T. J., Nielsen, J. E., McCammon, J. A. & Baker, N. A. (2004). *Nucleic Acids Res.* **32**, W665–W667. 10.1093/nar/gkh381 PMC441519 15215472

[bb18] Emsley, P. & Cowtan, K. (2004). *Acta Cryst.* D**60**, 2126–2132. 10.1107/S0907444904019158 15572765

[bb19] Emsley, P., Lohkamp, B., Scott, W. G. & Cowtan, K. (2010). *Acta Cryst.* D**66**, 486–501. 10.1107/S0907444910007493 PMC2852313 20383002

[bb20] Evans, P. R. (2011). *Acta Cryst.* D**67**, 282–292. 10.1107/S090744491003982X PMC3069743 21460446

[bb21] Evans, P. R. & Murshudov, G. N. (2013). *Acta Cryst.* D**69**, 1204–1214. 10.1107/S0907444913000061 PMC3689523 23793146

[bb22] Gehrig, S., Eberle, M. E., Botschen, F., Rimbach, K., Eberle, F., Eigenbrod, T., Kaiser, S., Holmes, W. M., Erdmann, V. A., Sprinzl, M., Bec, G., Keith, G., Dalpke, A. H. & Helm, M. (2012). *J. Exp. Med.* **209**, 225–233. 10.1084/jem.20111044 PMC3280868 22312113

[bb23] Griffiths, S., Byrne, R. T., Antson, A. A. & Whelan, F. (2012). *Acta Cryst.* F**68**, 333–336. 10.1107/S1744309112003831 PMC3310545 22442237

[bb24] Hendrickson, T. L. (2001). *Proc. Natl Acad. Sci. USA*, **98**, 13473–13475. 10.1073/pnas.251549298 PMC61062 11717415

[bb25] Hendrickson, W. A., Horton, J. R. & LeMaster, D. M. (1990). *EMBO J.* **9**, 1665–1672. 10.1002/j.1460-2075.1990.tb08287.x PMC551863 2184035

[bb26] Jühling, F., Mörl, M., Hartmann, R. K., Sprinzl, M., Stadler, P. F. & Pütz, J. (2009). *Nucleic Acids Res.* **37**, D159–D162. 10.1093/nar/gkn772 PMC2686557 18957446

[bb27] Kabsch, W. (2010). *Acta Cryst.* D**66**, 125–132. 10.1107/S0907444909047337 PMC2815665 20124692

[bb28] Kasprzak, J. M., Czerwoniec, A. & Bujnicki, J. M. (2012). *BMC Bioinformatics*, **13**, 153. 10.1186/1471-2105-13-153 PMC3674756 22741570

[bb29] Kato, T., Daigo, Y., Hayama, S., Ishikawa, N., Yamabuki, T., Ito, T., Miyamoto, M., Kondo, S. & Nakamura, Y. (2005). *Cancer Res.* **65**, 5638–5646. 10.1158/0008-5472.CAN-05-0600 15994936

[bb30] Kim, D. E., Chivian, D. & Baker, D. (2004). *Nucleic Acids Res.* **32**, W526–W531. 10.1093/nar/gkh468 PMC441606 15215442

[bb31] Krissinel, E. & Henrick, K. (2004). *Acta Cryst.* D**60**, 2256–2268. 10.1107/S0907444904026460 15572779

[bb32] Kuchino, Y. & Borek, E. (1978). *Nature (London)*, **271**, 126–129. 10.1038/271126a0 202873

[bb33] Landau, M., Mayrose, I., Rosenberg, Y., Glaser, F., Martz, E., Pupko, T. & Ben-Tal, N. (2005). *Nucleic Acids Res.* **33**, W299–W302. 10.1093/nar/gki370 PMC1160131 15980475

[bb34] Langer, G., Cohen, S. X., Lamzin, V. S. & Perrakis, A. (2008). *Nature Protoc.* **3**, 1171–1179. 10.1038/nprot.2008.91 PMC2582149 18600222

[bb35] Laskowski, R. A. & Swindells, M. B. (2011). *J. Chem. Inf. Model.* **51**, 2778–2786. 10.1021/ci200227u 21919503

[bb37] Matthews, B. W. (1968). *J. Mol. Biol.* **33**, 491–497. 10.1016/0022-2836(68)90205-2 5700707

[bb38] McCoy, A. J., Grosse-Kunstleve, R. W., Adams, P. D., Winn, M. D., Storoni, L. C. & Read, R. J. (2007). *J. Appl. Cryst.* **40**, 658–674. 10.1107/S0021889807021206 PMC2483472 19461840

[bb39] McNicholas, S., Potterton, E., Wilson, K. S. & Noble, M. E. M. (2011). *Acta Cryst.* D**67**, 386–394. 10.1107/S0907444911007281 PMC3069754 21460457

[bb40] Motorin, Y. & Helm, M. (2010). *Biochemistry*, **49**, 4934–4944. 10.1021/bi100408z 20459084

[bb41] Murshudov, G. N., Skubák, P., Lebedev, A. A., Pannu, N. S., Steiner, R. A., Nicholls, R. A., Winn, M. D., Long, F. & Vagin, A. A. (2011). *Acta Cryst.* D**67**, 355–367. 10.1107/S0907444911001314 PMC3069751 21460454

[bb42] Nallagatla, S. R., Jones, C. N., Ghosh, S. K., Sharma, S. D., Cameron, C. E., Spremulli, L. L. & Bevilacqua, P. C. (2013). *PLoS One*, **8**, e57905. 10.1371/journal.pone.0057905 PMC3587421 23483938

[bb43] Newman, J. (2004). *Acta Cryst.* D**60**, 610–612. 10.1107/S0907444903029640 14993709

[bb44] Newman, J., Egan, D., Walter, T. S., Meged, R., Berry, I., Ben Jelloul, M., Sussman, J. L., Stuart, D. I. & Perrakis, A. (2005). *Acta Cryst.* D**61**, 1426–1431. 10.1107/S0907444905024984 16204897

[bb45] Painter, J. & Merritt, E. A. (2006). *Acta Cryst.* D**62**, 439–450. 10.1107/S0907444906005270 16552146

[bb46] Park, F., Gajiwala, K., Noland, B., Wu, L., He, D., Molinari, J., Loomis, K., Pagarigan, B., Kearins, P., Christopher, J., Peat, T., Badger, J., Hendle, J., Lin, J. & Buchanan, S. (2004). *Proteins*, **55**, 772–774. 10.1002/prot.20086 15103641

[bb47] Rakovich, T., Boland, C., Bernstein, I., Chikwana, V. M., Iwata-Reuyl, D. & Kelly, V. P. (2011). *J. Biol. Chem.* **286**, 19354–19363. 10.1074/jbc.M111.219576 PMC3103313 21487017

[bb48] Rider, L. W., Ottosen, M. B., Gattis, S. G. & Palfey, B. A. (2009). *J. Biol. Chem.* **284**, 10324–10333. 10.1074/jbc.M806137200 PMC2667719 19139092

[bb49] Robert, X. & Gouet, P. (2014). *Nucleic Acids Res.* **42**, W320–W324. 10.1093/nar/gku316 PMC4086106 24753421

[bb51] Sheldrick, G. M. (2008). *Acta Cryst.* A**64**, 112–122. 10.1107/S0108767307043930 18156677

[bb52] Söding, J., Biegert, A. & Lupas, A. N. (2005). *Nucleic Acids Res.* **33**, W244–W248. 10.1093/nar/gki408 PMC1160169 15980461

[bb53] Spinola, M., Galvan, A., Pignatiello, C., Conti, B., Pastorino, U., Nicander, B., Paroni, R. & Dragani, T. A. (2005). *Oncogene*, **24**, 5502–5509. 10.1038/sj.onc.1208687 15870694

[bb54] Stivala, A., Wybrow, M., Wirth, A., Whisstock, J. C. & Stuckey, P. J. (2011). *Bioinformatics*, **27**, 3315–3316. 10.1093/bioinformatics/btr575 21994221

[bb55] Terwilliger, T. C. (2000). *Acta Cryst.* D**56**, 965–972. 10.1107/S0907444900005072 PMC2792768 10944333

[bb56] Terwilliger, T. C., DiMaio, F., Read, R. J., Baker, D., Bunkóczi, G., Adams, P. D., Grosse-Kunstleve, R. W., Afonine, P. V. & Echols, N. (2012). *J. Struct. Funct. Genomics*, **13**, 81–90. 10.1007/s10969-012-9129-3 PMC3375004 22418934

[bb57] Terwilliger, T. C., Grosse-Kunstleve, R. W., Afonine, P. V., Moriarty, N. W., Zwart, P. H., Hung, L.-W., Read, R. J. & Adams, P. D. (2008). *Acta Cryst.* D**64**, 61–69. 10.1107/S090744490705024X PMC2394820 18094468

[bb58] Vagin, A. & Teplyakov, A. (2010). *Acta Cryst.* D**66**, 22–25. 10.1107/S0907444909042589 20057045

[bb59] Xing, F., Hiley, S. L., Hughes, T. R. & Phizicky, E. M. (2004). *J. Biol. Chem.* **279**, 17850–17860. 10.1074/jbc.M401221200 14970222

[bb60] Yu, F., Tanaka, Y., Yamashita, K., Suzuki, T., Nakamura, A., Hirano, N., Suzuki, T., Yao, M. & Tanaka, I. (2011). *Proc. Natl Acad. Sci. USA*, **108**, 19593–19598. 10.1073/pnas.1112352108 PMC3241823 22123979

[bb61] Zinshteyn, B. & Gilbert, W. V. (2013). *PLoS Genet.* **9**, e1003675. 10.1371/journal.pgen.1003675 PMC3731203 23935536

