# Supplementary material for: From bacterial to human dihydrouridine synthase: automated structure determination
Source: Acta Crystallogr D Biol Crystallogr. 2015 Jun 30;71(Pt 7):1564–71. doi: 10.1107/S1399004715009220 (PMC4498606; doi:10.1107/S1399004715009220)
Supplement: Supplementary file 2 [file d-71-01564-sup1.pdf]

BIOLOGICAL
CRYSTALLOGRAPHY

Volume 71 (2015)

Supporting information for article:

From bacterial to human dihydrouridine synthase: automated structure determination

Fiona Whelan, Huw T. Jenkins, Sam C. Griffiths, Robert T. Byrne, Eleanor J. Dodson and Alfred A. Antson

Table S1 Top scoring *phenix.mr_rosetta* placed models and map correlation between *2mFo-DFc* maps calculated from placed models and the final refined structure of hDUS2.

Search model	*Phaser* TF Z-score/LLG	Map correlation
*Tt*Dus (3b0p)	4.7/39.7	0.36
*Tm*Dus (1vhn)	5.2/26.0	0.35
*Ec*DusC (4bfa)	4.8/25.6	0.31

Table S2 Comparison of *Buccaneer* automatic model building into maps generated by *phenix.mr_rosetta* starting from structures of *Tt*Dus, *Tm*Dus and *Ec*DusC; and a comparison of *Buccaneer* built models obtained from *phenix.mr_rosetta* and from Se-SAD phasing.

Search model	*Buccaneer* sequenced residues (longest chain)	*Buccaneer* R/R_{free}	Cα rmsd (Å) † to *Buccaneer* model from Se-SAD (no. aligned)
*Tt*Dus (3b0p)	320 (320)	0.257/0.303	0.298 (295)
*Tm*Dus (1vhn)	322 (322)	0.255/0.301	0.305 (295)
*Ec*DusC (4bfa)	132 (51)	0.476/0.551	N/A
*Ec*DusC (4bfa)*	330 (330)	0.249/0.297	0.312(295)

* Phenix-dev-1980/Rosetta-2015_09_57646

† residues aligned: 7–87, 90–116, 136–188, 194–214, 223–336

Table S3 Statistics for a conventional molecular replacement approach, showing comparison of search models, *2mFo-DFc* map correlation of MR solutions with final refined hDUS2 structure, initial model refinement and *Buccaneer* autobuilding results.

Search model (catalytic domain)	Cα RMSD (Å) to final hDUS2 structure (no. residues aligned)	Sequence identity (after *HHpred* alignment)	*Phaser* TF Z-score /LLG	Map correlation	Results of *Refmac* jelly body refinement		*Buccaneer* Final R/Rfree
					R/Rfree	Map correlation	
*Tt*Dus (3b0p) 3–240	1.5 (203)	21.2	8.4/83.4	0.37	46.4/48.3	0.56	25.9/29.6
*Tm*Dus (1vhn) 5–228	2.0 (195)	21.9	5.2/25.0	0.32	47.4/49.2	0.48	N/A
*Ec*DusC (4bfa) 1–240	1.9 (200)	22.7	4.5/26.9	0.30	54.0/55.0	0.38	N/A
